# Incisions Made in the Skin of the Face

**Published:** 1897-06

**Authors:** 


INCISIONS made in the skin of the face should be placed where there is a shadow, or in the bottom of the furrows produced by the habitual expression of the patient. When this cannot be done, the incision should be parallel to the facial line, rather than across it. A slightly curved incision makes a less conspicuous scar than the straight or abruptly curved one.—*Railway Surgeon.*

